# Changes in the expression levels of elastic fibres in yak lungs at different growth stages

**DOI:** 10.1186/s12861-021-00240-w

**Published:** 2021-04-20

**Authors:** Jingyi Li, Xiangqiong Meng, Lihan Wang, Yang Yu, Hongxian Yu, Qing Wei

**Affiliations:** 1 College of Eco-Environmental Engineering, Qinghai University, 251 Ningda Road, Xining, 810016 Qinghai China; 2 Qinghai Academy of Animal Science and Veterinary Medicine, Qinghai University, 1 Weier Road, Xining, 810016 Qinghai China; 3 Department of Veterinary Medicine, College of Agriculture and Animal Husbandry, Qinghai University, 251 Ningda Road, Xining, 810016 Qinghai China; 4 State Key Laboratory of Plateau Ecology and Agriculture, Qinghai University, 251 Ningda Road, Xining, 810016 Qinghai China

**Keywords:** Yak, Lung tissue, Elastic fibres, Development, Transcriptome

## Abstract

**Background:**

Yaks have a strong adaptability to the plateau environment, which can be attributed to the effective oxygen utilization rate of their lung tissue. Elastic fibre confers an important adaptive structure to the alveolar tissues in yaks. However, little research has been focused on the structural development of lung tissues and the expression levels of elastic fibres in yaks after birth. Therefore, this study aimed to investigate the morphological changes of elastic fibers and expression profiles of fibre-formation genes in yak lungs at different growth stages and the relationship between these changes and plateau adaptation.

**Results:**

Histological staining was employed to observe the morphological changes in the lung tissue structure of yaks at four different ages: 1 day old, 30 days old, 180 days old and adult. There was no significant difference in the area of a single alveolus between the 1-day-old and 30-day-old groups (*P*-value > 0.05). However, the single alveolar area was gradually increased with an increase in age (*P*-value < 0.05). Elastic fibre staining revealed that the amount of elastic fibres in alveolar tissue was increased significantly from the ages of 30 days to 180 days (*P*-value < 0.05) and stabilized during the adult stage. Transcriptome analysis indicated that the highest levels of differentially expressed genes were found between 30 days of age and 180 days of age. KEGG analysis showed that PI3K-Akt signalling pathway and MAPK pathway, which are involved in fibre formation, accounted for the largest proportion of differentially expressed genes between 30 days of age and 180 days of age. The expression levels of 36 genes related to elastic fibre formation and collagen fibre formation were also analysed, and most of these genes were highly expressed in 30-day-old and 180-day-old yaks.

**Conclusions:**

The content of elastic fibres in the alveolar tissue of yaks increases significantly after birth, but this change occurs only from 30 days of age to 180 days of age. Our study indicates that elastic fibres can improve the efficiency of oxygen utilization in yaks under harsh environmental conditions.

## Background

Yak is the only bovine animal that can grow and reproduce in the arctic-alpine pastoral area of the Qinghai-Tibet Plateau. This animal has strong adaptability to its ecological environment, and can tolerate harsh environmental conditions such as hypoxia, cold and short herbage growing periods. Yak is important to the animal husbandry of the Qinghai-Tibetan Plateau and is an essential means of livelihood and production for local people, as being well-known as the “ship of the plateau” and “all-round livestock” [1]. With the continuous development of yak resources, sustainable yak production has become the highest priority of animal husbandry in plateau areas.

After a long period of natural and artificial selection, yak has developed unique morphological, physiological and hereditary traits that are different from other bovine animals [2]. As the main respiratory organ, lung tissue is an important functional organ for animals to adapt to the external environment. The yak inhales oxygen from the external environment through the lung tissue, and further provides the body with oxygen molecules through the gas exchange system. Yak has attracted wide attention from scholars in this country and abroad, due to its good adaptability to plateau environments. In recent years, several studies have assessed the morphological structures of organs and tissues in adult yaks [3]. Anatomically, yak ribs are relatively long and the intercostal spacing is large, which can increase the chest size and provide a large space for the development of the heart and lungs. The large diameter of the yak windpipe can increase the amount of air enters the body [4]. Histologically, the yak trachea is rich in goblet cells, the alveolar diaphragm is thick, the pulmonary arterioles are thin, and the gas-blood barrier is relatively thin, which can be conducive to the passage and diffusion of oxygen [5]. In the yak cardiovascular system, enhancing the conduction of cardiac excitation through conductive fibres can increase the length and density of capillaries in the heart, thereby increasing oxygen delivery [6]. With regard to skeletal muscle histology, the diameter of yak muscle fibre is relatively small, thus increasing the density of muscle fibre per unit area. In addition, the content of elastic fibres in yak muscle is relatively rich, which effectively improves its adaptability to hypoxia [7]. Physiologically, the red blood cell number and haemoglobin content of yaks are relatively high, and these values increase with increasing altitudes, which in turn helps to promote the efficiency of oxygen transport in the blood [8]. However, little research has been focused on the structural development of lung tissues and the expression levels of elastic fibres in yaks after birth.

Elastic fibres have many branches and are widely distributed, which can be interwoven into a net and arranged into a film in lung tissue. The elastic membranes are alternately combined to form elastic membrane units, or known as elastic arterial resilience units [9, 10]. Previous studies have demonstrated that mature elastic fibres and elastic membranes are composed of homologous elastin macromolecules that form scaffolds along microfibrils arranged in parallel [11, 12]. Because of the presence of elastic and collagen fibers in the parenchyma, which are beneficial for gas exchange, the lungs have good elasticity [13]. Therefore, we investigated the morphological changes of elastic fibres in yak lung tissues at different growth stages from the perspective of histological observation. The mechanism governing fibre formation was further elucidated by transcriptome analysis, in order to provide new insights into the molecular mechanisms underlying yak adaptation to hypoxia and establish a foundation for future research in plateau medicine and other disciplines.

## Results

### Histological observation of yak alveolar tissues at different growth stages

**Fig. 1:**
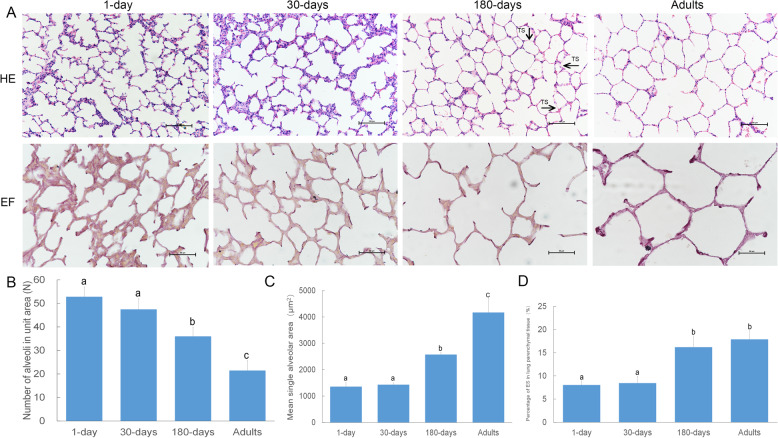
Basic structure and quantitative measurement of alveolar tissue in yaks at different growth stages. (**a**) HE staining and elastic fibre staining of the alveolar tissues of plateau yaks at four ages: 1 day old, 30 days old, 180 days old and adult. The number of alveoli per unit area (**b**), average single alveolar area (**c**), and percentage of elastic fibres (**d**) in the lung parenchyma of yaks. Note: TS: translucent structure

The results showed that the morphologies of lung alveoli in yak were similar at different ages, and most of the alveoli were irregular oblate and oval. Different alveolar sizes were observed in 1-day-old yaks, which were smaller than those of 30-day-old, 180-day-old and adult yaks. The elastic fibres were determined to be evenly distributed in the alveolar septum, while those at the top of the alveolar septum were inequitably distributed. Some translucent structures could be observed in the alveoli of 180-day-old yaks, and the number of elastic fibres was increased significantly (Fig. 1a). Based on the quantitative data analysis (Fig. 1b), the number of alveoli per unit area was not significantly different between the 1-day-old and 30-day-old yaks (*P*-value > 0.05), but differed significantly between the 30-day-old and adult yaks (*P* < 0.05). The average single alveolar area (Fig. 1c) was not significant different between the 1-day-old and 30-day-old yaks (*P*-value > 0.05), but it gradually increased from the 30-day-old yak to the adult yak (*P*-value < 0.05). The percentage of elastic fibres in the lung parenchyma showed an increasing trend (Fig. 1d) from the 30-day-old yak to the 180-day-old yak (*P*-value < 0.05), but there were no significant differences between the 1-day-old and 30-day-old yaks (*P*-value > 0.05) as well as the 180-day-old and adult yaks (*P*-value > 0.05).

### Transcriptome analysis

**Fig. 2:**
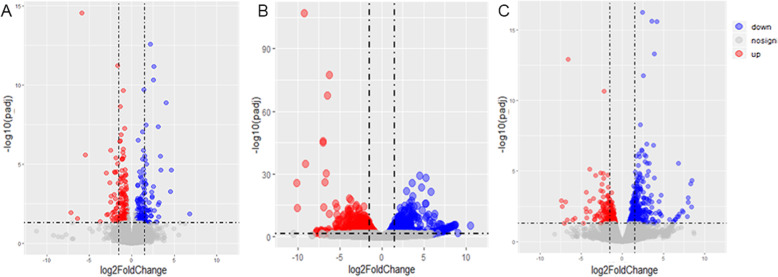
Differentially expressed genes in volcanic maps. (**a**) One day old vs. 30 days old; (**b**) 30 days old vs. 180 days old; and (**c**) 180 days old vs. adult yaks

The difference in gene expression between two age groups was analysed by differential expression analysis software (DESeq2), and the test parameters were |Fold-change| (multiple difference) > 1.5, *p*-value < 0.05. The results showed that there were 17,218 genes (69.71%) expressed at 1 day of age vs. 30 days of age, and 1.28% (317) of these genes were differentially expressed, including 142 upregulated genes (0.57%) and 175 downregulated genes (0.71%). A total of 17,404 (70.47%) genes were expressed at 30 days of age vs. 180 days of age, and 3190 (12.92%) genes were differentially expressed, including 1775 (7.19%) upregulated genes and 1415 (5.73%) downregulated genes. Besides, 17,232 (69.77%) genes were expressed in 180-day-old yaks vs. adult yaks, with 695 (2.81%) differentially expressed genes, of which 425 (1.72%) were upregulated and 270 (1.09%) were downregulated. Overall, the differentially expressed genes between 30-day-old yaks and 180-day-old yaks were the most abundant (Table 1 and Fig. 2).

**Table 1:** Analysis results of differentially expressed genes

Age	Total expressed gene	Differentially expressed gene	Up-regulated gene	Down-regulated gene
**1-day-old vs. 30-day-old**	17,218	317	142	175
**30-day-old vs. 180-day-old**	17,404	3190	1775	1415
**180-day-old vs. adult**	17,232	695	425	270

### GO analysis of differentially expressed genes

**Fig. 3:**
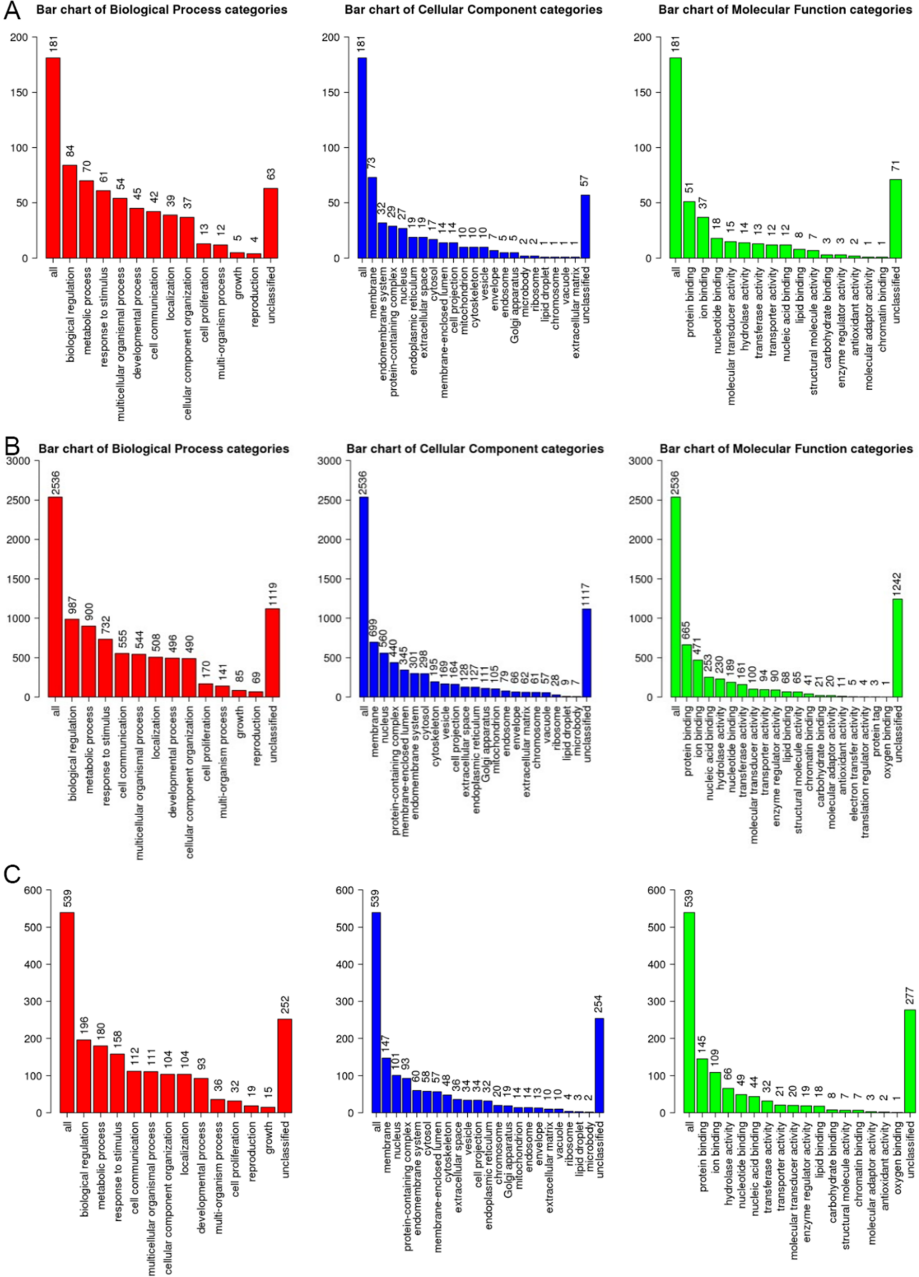
GO enrichment analysis of differentially expressed genes. (**a**) One day old vs. 30 days old; (**b**) 30 days old vs. 180 days old; and (**c**) 180 days old vs. adult yaks

Through GO enrichment analysis (Fig. 3), the differentially expressed genes of 1-day-old yaks vs. 30-day-old yaks, 30-day-old yaks vs. 180-day-old yaks, and 180-day-old yaks vs. adult yaks were analysed. The main biological processes (BP) associated with differential gene expression in each group include developmental processes and stimulus stress. The cell composition (CC) mainly concentrates on several membrane and intimal systems. The molecular function (MF) mainly involves the functions of protein binding and ion binding.

### KEGG pathway analysis

**Fig. 4:**
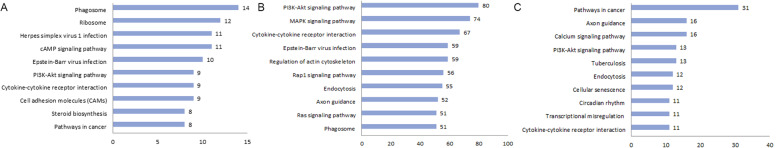
KEGG pathway analysis of differentially expressed genes. (**a**) One day old vs. 30 days old; (**b**) 30 days old vs. 180 days old; and (**c**) 180 days old vs. adult yaks. Only the top 10 pathways are listed

KEGG analysis was performed to determine the main biochemical metabolic pathways and signal transduction pathways associated with the differentially expressed genes. The results demonstrated that PI3K-Akt signalling pathway was mapped for the differentially expressed genes between two adjacent ages (Fig. 4). In the 30 days old vs. 180 days old yak groups, PI3K-Akt signalling pathway was the most important pathway associated with differential gene expression, followed by MAPK signalling pathway (Fig. 4b).

### Screening of fibrogenic genes

By reviewing the relevant studies conducted both in this country and abroad and combining them with GO annotation, we identified 36 genes involved in fibre production (Table 2). Among these genes, 22 (61.11%) and 14 (38.89%) were upregulated and downregulated, respectively. Two genes were differentially expressed between 1-day-old and 30-day-old yaks, while 34 genes were differentially expressed between 30-day-old and 180-day-old yaks. However, there was no significant difference in the expression levels of these genes between 180-day-old and adult yaks.

**Table 2:** Annotation of genes related to fiber formation

Gene name	KEGG ID	Description
COL3A1	102,279,325	Collagen type III alpha 1
COL11A1	102,276,187	Collagen type XI alpha 1
COL11A2	102,285,415	Collagen type XI alpha 2
COL1A2	102,267,202	Collagen type I alpha 2
ADAMTS2	102,285,627	A disintegrin and metalloproteinase with thrombospondin motifs 2
LOX	102,276,831	Lysyl oxidase
LOXL2	102,282,891	Lysyl oxidase like 2
VIL1	102,272,630	Villin 1
PHACTR2	102,278,950	Phosphatase and actin regulator 2
TGFβ1	102,283,357	Transforming growth factor beta 1
ACAN	102,278,312	Aggrecan
TGFβ2	102,283,491	Transforming growth factor beta 2
TGFBI	102,284,294	Transformed growth factor beta induced (68 kDa)
CAMSAP3	102,274,353	Calmodulin regulated spectrin associated protein family member 3
CDC42BPA	102,284,687	CDC42 binding protein kinase alpha
BAIAP2	102,286,151	BAI1 associated protein 2
RASAL3	102,269,674	RAS protein activator 3
ITGB1	102,273,972	Integrin subunit beta1
DNM2	102,279,431	Motor protein 2
STIM1	102,271,938	Matrix interacting molecule 1
RNF44	102,276,069	Ring finger protein 44
NDRG1	102,266,961	N-Myc downstream-regulated gene 1
FGF1	102,287,352	Fibroblast growth factor 1
FGF9	102,273,668	Fibroblast growth factor 9
FGF18	102,287,270	Fibroblast growth factor 18
FIBP	102,276,075	FGF1 intracellular binding protein
CNPY3	102,287,727	Canopy FGF signalling regulator 3
TLR3	102,268,437	Toll-like receptor 3
FN1	102,280,180	Fibronectin 1
FAM65B	102,273,075	Family with sequence similarity 65 member B
GPX1	102,280,278	Glutathione peroxidase 1
FBN1	102,283,369	Fibrin 1
FBN2	102,267,459	Fibrin 2
EMILIN3	102,288,344	Elastin microfiber junction 3
EMILIN2	102,271,699	Elastin microfiber junction 2
ELN	106,700,709	Elastin

### Expression patterns of fibrogenesis-related genes at different growth stages

**Fig. 5:**
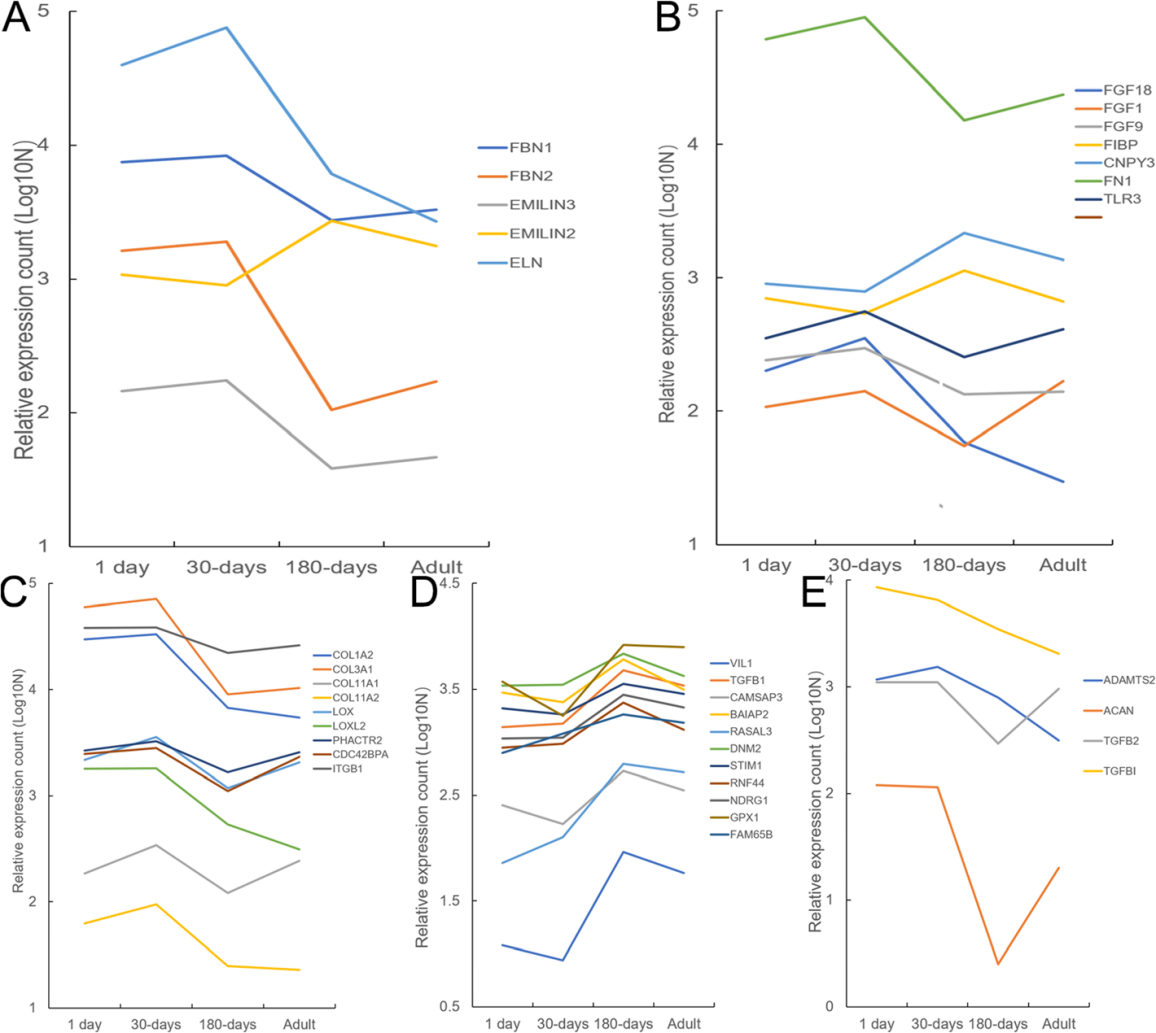
Changes in the expression levels of fibre formation-related genes in yaks at different growth stages. (**a**) Elastic fibre formation-related genes; (**b**) fibroblast-related genes; (**c**) expression levels of collagen fibre formation-promoting genes reached a maximum at 30 days of age; (**d**) expression levels of collagen fibre formation-promoting genes reached a maximum at 180 days of age; and (**e**) collagen fibre formation-inhibiting genes

Five elastic fibre formation-related genes were selected, which have been previously reported to promote fibre formation. The expression levels of these genes in yak lung tissues at different growth stages were analysed, and the results showed that these genes were highly expressed at 30 days of age or 180 days of age (Fig. 5a). Moreover, seven genes related to fibroblasts were selected, and their functions may also promote fibrogenesis. The expression levels of these genes were remarkably upregulated in the 30-day-old and 180-day-old yak groups (Fig. 5b). Furthermore, 24 genes related to collagen fibre formation were selected, among which 20 genes could promote fibre formation. The expression levels of 9 genes were the highest at 30 days of age (Fig. 5c), while 11 genes were the highest at 180 days of age (Fig. 5d). However, 4 genes had inhibitory effects on fibrogenesis, and their expression levels were all decreased after 30 days of age (Fig. 5e).

## Discussion

### Histological observation of yak alveolar tissues at different growth stages

The alveolus is a functional unit of the lungs, and the efficiency of gas exchange is largely dependent on the size of respiratory area in the lungs [14]. With continuous growth and development, the lung volume and surface area of yaks increase, the number of alveoli per unit area decreases, and the total number of alveoli increases (Fig. 1). This may expand the area of gas exchange in the lungs, accelerate the rate of gas exchange in lung tissue [15] and improve the utilization rate of oxygen, thus enabling yaks to adapt quickly to low-oxygen and high-altitude environments. The ability of yaks to tolerate low oxygen at high altitudes has considerable significance [16]. Elastic fibers play an important role in the development of lung tissue. These fibers provide a strong retraction force for yaks to exchange air between the outside atmosphere and the blood in the lungs, and enable blood vessels to bear the pressure of the heartbeats, thereby maintaining a constant blood flow [17]. In this study, we found that the amount of elastic fibers in the alveoli continued to increase, especially after 180 days of age. At the same time, after HE staining and morphological analysis, we also observed the “transparent membrane” in the lung alveoli at 180 days of age as mentioned in the previous reports [18]. Based on the morphological features of elastic fibers at different developmental stages, we speculate that the absence of cell structures in the alveolar septum and the formation of transparent membranes can reflect the abundant number of elastic fibers in the lung tissues. Moreover, the performance of this structure at 180 days of age is similar to that of the adult group, which also reflects that the lung tissue of the yak is close to mature at the age of 180 days.

### Expression profiles of differentially expressed genes

By distinguishing the biological information of transcriptomic data between two age groups, it was found that the comparison between 30-day-old and 180-day-old groups yielded the highest number of differentially expressed genes (Fig. 2b). Moreover, the growth stage from 30 days old to 180 days old involved many gene expression changes, as this period is a critical stage of lung tissue development in yaks. These results were consistent with our previous morphological observations.

### GO and KEGG annotations

GO enrichment analysis showed that the differentially expressed genes in three age groups were associated with development process, mainly in the membrane and nucleus. The main biological processes were biological regulation and metabolism, and the process involved the functions of protein binding and ion binding. The proportion of differentially expressed genes in yak lung tissue was highest between the 30-day-old and 180-day-old groups (Fig. 3b). It is speculated that 30–180 days of age for yak is the critical period of yak development. It is speculated that 30–180 days old of yak is the key period of yak development. In addition to the participation of a large number of genes related to development, other genes related to growth and stress have also appeared, and also involved in many different biological processes, which may be related to the special environment of the plateau where the yak lives. KEGG pathway analysis showed that PI3K-Akt signalling pathway was an important cellular regulation pathway in three age groups and was related to the formation of elastic fibres [19]. The signalling pathways involved in the formation of fibres were mostly observed in both 30-day-old and 180-day-old groups [20]. Among these pathways, PI3K-Akt signalling pathway accounted for the largest proportion (Fig. 4b), followed by MAPK, which was also closely related to cell growth and development [21].

### Genes related to fibrogenesis

Thirty-four of the 36 genes involved in fibre formation were differentially expressed from 30 days of age to 180 days of age, indicating that a large number of elastic and collagen fibres has been formed at this stage. Moreover, the expression levels of fibrogenesis-promoting genes were significantly increased between 30-day-old and 180-day-old groups (*P*-value < 0.05), while those of fibrogenesis-inhibiting genes were decreased in the two groups. The genes related to elastic fibrogenesis are *FBN1*, *FBN2*, *EMILIN3*, *EMILIN2* and *ELN*, of which *FBN1* and *FBN2* belong to the fibrillin protein family [22] and *EMILIN3*, *EMILIN2* and *ELN* belong to the elastin family. The fibrillin and elastin family genes are closely related to the formation of elastic fibres [23]; therefore, we selected *ELN* for further analysis. It was found that the expression level of *ELN* reached its maximum at 30 days of age (Fig. 5a), and this gene was highly expressed in lung tissue. Elastic fibre is a stretched rubber-like fibre that can provide elasticity and tensile strength to lung tissues. Elastin is most abundant in elastic fibres, and its core is surrounded by a mantle of glycoprotein- and fibrillin-rich microfibrils, which are necessary to maintain the integrity of elastic fibres [24]. Although collagen can exhibit strength and toughness in the extracellular matrix, it needs to be elastic for lung tissue, and the elasticity primarily depends on elastic fibres in the extracellular matrix.

There are 7 fibroblast-related genes (*FGF1*, *FGF9*, *FGF18*, *FIBP*, *CNPY3*, *TLR3* and *FN1*) that can promote the formation of fibroblasts. Fibroblast growth factors have a wide range of biological activities, including cell proliferation and differentiation [25]. These growth factors can promote the mitosis of fibroblasts and growth of mesodermal cells, stimulate the formation of blood vessels, and play major roles in wound healing and limb regeneration [26]. Fibroblast-related genes can promote the growth of fibroblasts and subsequently cause them to develop into fibroblasts [27]. The expression levels of fibroblast-related genes reached a maximum at 30 or 180 days of age (Fig. 5b), suggesting that it is the main formation stage of elastic fibers.

There are 20 genes that can promote the formation of collagen fibres. The collagen family is primarily associated with cell composition, and other related genes mainly participate in fibre formation by inducing various cytokines and growth factors [28]. In this study, the expression level of *COL3A1* reached the highest value at 30 days of age (Fig. 5c). This type III collagen can keep the skin firm and elastic, promote the migration, differentiation and proliferation of cells, and induce the production of collagen fibres [29]. Moreover, the expression level of *GPX1* reached its maximum at 180 days of age (Fig. 5d), indicating that glutathione peroxidase can enhance the formation of collagen fibres. Apart from this, glutathione peroxidase can improve the survival rate of cells and ensure the integrity of genetic DNA [30, 31]. Four genes (*ADAMTS2*, *ACAN*, *TGFβ2* and *TGFβ1*) could repress the formation of collagen fibres. These genes are mainly involved in the induction (Fig. 5e) or suppression of growth factors and cytokines prior to fibre formation.

## Conclusions

In this study, the elastic fibers in the lung tissue of yaks reached the highest expression level at 30–180 days of age, indicating that this stage is a critical period for the development of lung tissues in yaks. For example, during the development process (from 30 days old to 180 days old), the differentially expressed genes were at the highest levels, PI3K-Akt signalling pathway and MAPK pathway accounted for the largest proportion, and the genes related to fiber formation were also highly expressed. To improve the efficiency of oxygen utilization in the plateau environment, the amount of elastic fibers in the alveolar tissue of yaks was increased significantly from 30 to 180 days of age, and stabilized after 180 days. From the development trend of the number of elastic fibers, it is predicted that the critical period of the development and change of the yak lung tissue. The elastin in the elastic fibers of lung tissue can facilitate the recoil responses of the trachea, alveoli and vascular tubes. Hence, the trachea and pulmonary arteries have good dilatability and contractility, which in turn increases the elasticity of lung parenchyma, accelerates the rate of gas exchange, improves the efficiency of oxygen utilization and enables yaks to better adapt to the plateau environment. But adapting to hypoxic environment is a complicated physiological process, and its mechanism needs further study.

## Methods

### Experimental animals

One day old, 30 days old, 180 days old and adult yaks (3–4 years old) were purchased from herders of Haiyan area (3200 m above sea level), Qinghai province, China. The respiratory systems of these yaks were healthy, regardless of sex. All yaks were anesthetized with pentobarbital sodium (200 mg/kg; intravenous injection), and then killed by exsanguination through the abdominal aorta in a slaughter house. This experiment was performed according to the Animal Ethics Procedures and Guidelines of the People’s Republic of China.

### Histological staining

#### Paraffin sections of lung tissues

Fresh tissues were collected, fixed with 4% paraformaldehyde for 24 h, dehydrated with gradient alcohol, cleared with xylene, embedded in paraffin wax, sectioned at a thickness of 4 μm, and placed on glass slides for later use.

### Hematoxylin and eosin staining

For HE staining of tissue samples, reverse gradient alcohol rehydration was conducted followed by staining with haematoxylin for 5 min. After differentiation with diluted hydrochloric acid, the tissues were rinsed with running water, treated with 0.6% ammonia water until they turned blue, and rinsed again with running water. Finally, the tissues were stained with eosin for 1–3 min, and sealed through gradient alcohol dehydration.

### Elastic fibre staining

Tissue samples were subjected to reverse gradient alcohol rehydration, and then placed in Wiegert’s stain for 5 min. After washing with Wiegert bleach for 1 ~ 2 min, the tissues were differentiated with acidic differentiation solution for 2 ~ 3 s, rinsed with running water for 10 min, and re-dyed with VG staining solution for 30 s. Finally, the samples were sealed through gradient alcohol dehydration.

### Observation and measurement

The HE-stained and elastic fibre sections were examined using an Olympus BX51 microscope, and the images were captured and analysed with Image-Pro Plus 6.0. The area of single alveoli and the number of alveoli per unit area in HE-stained sections were measured; while the areas of lung parenchyma and elastic fibres at different growth stages were also measured. Excel was used to calculate the proportion of elastic fibres in alveolar tissue. SPSS 19.0 was used to perform statistical analysis among multiple groups. All data are expressed as mean ± standard deviation (SD). A *p*-value of < 0.05 was deemed as statistical significance.

### Transcriptome data analysis

#### Transcriptome sequencing

Total RNA was extracted from the lung tissue samples of 1-day-old, 30-day-old, 180-day-old and adult yaks using the TRIzol reagent (Invitrogen, USA) and then genomic DNA was eliminated by DNase I (Takara, Japan) according to the instructions. The quantity and quality of RNA were determined by measuring the OD260/280 and OD260 with NanoPhotometer NP80 (Implen, Germany). Equal amounts of high quality total RNA from the lung tissue of individual were then pooled to construct a library for RNA-seq analysis. cDNA library construction and sequencing were performed by Shanghai Liebing Biomedical Technology Co., Ltd. Sequencing was performed the NovaSeq sequencing platform by adopting pair-end sequencing mode. Briefly, mRNA with poly(A) were isolated from the total RNA using oligo (dT) beads, purified, fragmented (100 bp ~ 400 bp) with an ultra-sonicator and reverse transcribed into first strand cDNA using random primers. Subsequently, sequencing adapters were connected to the short fragments, and the resultant cDNA libraries used for paired-end RNA-seq. After the sample is tested, the eukaryotic mRNA is enriched with magnetic beads with Oligo (dT). Subsequently, the fragmentation buffer was used to break the mRNA into short fragments. Using mRNA as a template, a single-strand cDNA was synthesized using random hexamers, and then double-stranded cDNA was synthesized by adding buffer, dNTPs and DNA polymerase I and RNase H. The double-stranded cDNA was purified again using AMPure XP beads. The purified double-stranded cDNA was firstly end-repaired, A-tailed and ligated to the sequencing linker, and AMPure XP beads were used for fragment size selection. Finally, PCR amplification was performed and the PCR product was purified using AMPure XP beads to obtain the final library. After the library was constructed, preliminary quantification was performed using Qubit2.0, and the library was diluted to 1.5 ng/ul. Then, the insert size of the library was detected using Agilent 2100. After the insert size was as expected, the effective concentration of the library was determined by Q-PCR method. Accurate quantification (library effective concentration > 2 nM) was performed to ensure library quality. After the library was qualified, the different libraries were pooled according to the effective concentration and the target data volume, and then was sequenced.

### Bioinformatic analysis

After obtaining clean reads, we use Trinity (Grabherretal, 2011) to stitch clean reads to obtain reference sequences for subsequent analysis. The NovelBio Annotation platform is used for gene annotation, and we use Hisat2 software to align and splice RNA sequences. Gene Ontology (GO) and Kyoto Encyclopedia of Genes and Genomes (KEGG) databases were used to analyse the obtained and verified transcriptomic data. Differential expression analysis of different groups was performed using the DESeq R package (1.10.1). DESeq provide statistical routines for determining differential expression in digital gene expression data using a model based on the negative binomial distribution. The resulting *P* values were adjusted using the Benjamini and Hochberg’s approach for controlling the false discovery rate. To screen differentially expressed genes, the parameters of 1.5 times the difference and false discovery rate (FDR) ≤ 0.05 were applied. Identification and quantification of fibre generation-related genes in yak samples at different growth stages were then performed. The change in fibrogenic gene expression patterns at each period was measured and analysed.

## Data Availability

The datasets generated and analysed during the current study are available in the NCBI repository (http://www.ncbi.nlm.nih.gov/bioproject/719069), and RNA sequencing data has been deposited into NCBI bank (ID: 719069).

## Abbreviations

HE: Hematoxylin eosin
GO: Gene ontology
KEGG: Kyoto encyclopedia of genes and genomes
TS: Translucent structure
